# Identifying the Structure and Elements of Nutritional Guidance Techniques: Cross-Sectional Analytic Hierarchy Study

**DOI:** 10.2196/83185

**Published:** 2026-02-05

**Authors:** Machiko Ukai, Mikiko Kanno, Rui Sudo, Miyako Ogawa, Kazuki Ohashi, Katsuhiko Ogasawara

**Affiliations:** 1 Graduate School of Health Sciences, Hokkaido University, Sapporo, Japan; 2 Department of Nutrition, Teine Keijinkai Hospital, Sapporo, Japan; 3 Teine Family Medical Clinic, Sapporo, Japan; 4 College of Agriculture, Food and Environment Sciences, Rakuno Gakuen University, Ebetsu, Japan; 5 Faculty of Health Sciences, Hokkaido University, N12-W5, Kita-ku, Sapporo, 060-0812, Japan, 81 117063409; 6 Faculty of Engineering, Muroran Institute of Technology, Muroran, Japan

**Keywords:** nutrition counseling, dietitians and education, health education, educational models, decision-making, clinical competence, professional competence, medical informatics

## Abstract

**Background:**

Registered dietitian nutritionists (RDNs)—referred to as registered dietitians in Japan—contribute to disease management, prevention of complications, and improvement in quality of life through individualized nutritional guidance. However, these techniques often rely on individual experience, leading to variations in quality. The nutrition care process provides a standardized framework for nutritional care, but the specific techniques used in clinical practice and their interrelationships remain unclear. Interpretive structural modeling (ISM) is a method that visualizes and hierarchically organizes interrelationships among multiple elements, making it useful for structuring complex practical skills. Therefore, clarifying the structure of nutritional guidance techniques may support the standardization of practice and the development of educational frameworks.

**Objective:**

This study aimed to identify the elements influencing nutritional guidance techniques in clinical practice, clarify their hierarchical structure using ISM, and explore their potential applicability to the education of registered dietitians.

**Methods:**

Three experienced RDNs participated in an expert panel. Elements influencing nutritional guidance techniques were identified through structured brainstorming and consensus-building sessions. The extracted elements were analyzed using ISM to generate a reachability matrix and derive a hierarchical structure that visualized the interrelationships among the elements.

**Results:**

A total of 14 elements were identified and organized into a 6-level hierarchical structure. The upper levels included nutrition care process–related elements, with the “nutritional intervention plan” positioned at the top, whereas the lower levels consisted of foundational elements such as “clinical knowledge” and “understanding of patient background.”

**Conclusions:**

This study identified 14 elements influencing nutritional guidance techniques in clinical practice and systematically visualized their interrelationships as a 6-level hierarchy using ISM. The resulting model provides an initial framework that may inform the development of clinical education curricula and competency evaluation frameworks for RDNs, and it could contribute to the advancement of standardized approaches in nutritional guidance education.

## Introduction

In clinical settings, registered dietitian nutritionists (RDNs) play a critical role in delivering individualized nutritional guidance tailored to each patient, thereby contributing to disease management, prevention of complications, and improvement in quality of life, with their effectiveness being well documented [1-5].

With the growing public interest in nutrition, the significance of RDNs is increasingly recognized. To provide systematic and evidence-based nutritional care, the nutrition care process (NCP) has been internationally standardized, and its framework is defined by the NCP model [6,7].

Although the NCP offers a standardized procedural framework for nutritional care, it does not capture the specific techniques used by dietitians in practice or the interrelationships among them. In clinical nutrition counseling, not only professional knowledge but also skills such as communication, patient motivation, value sharing, and understanding of the patient background are essential [8]. These elements are often dependent on the individual dietitian’s experience and skill set, which can result in variability in the quality of nutritional counseling [9].

While previous studies have explored the competencies of expert dietitians [10-13] and conceptually defined their professional expertise [14], the specific components and interrelationships of nutritional guidance techniques in clinical practice remain unclear. To clarify the complex interplay among these techniques, it is necessary to move beyond anecdotal discussion and adopt a systematic and objective analytical method.

Interpretive structural modeling (ISM) [15] is a methodology designed to visualize and hierarchically structure interdependent relationships among elements. ISM has been applied in diverse fields, such as organizational theory, curriculum development, and medical informatics [16-20]. Its strength lies in structuring complex insights derived from expert knowledge, offering a valuable foundation for educational design that facilitates efficient learning for novices.

Accordingly, this study identified and visualized the hierarchical structure of elements influencing nutritional guidance techniques in clinical practice using the ISM approach. The findings are expected to contribute to the standardization of clinical nutrition counseling, the development of structured educational programs for dietitians, and the establishment of competency evaluation frameworks, ultimately enhancing the quality of clinical education.

## Methods

### Study Design and Participants

This study used a cross-sectional design using ISM to visualize the structural relationships among elements influencing nutritional guidance techniques in clinical practice. ISM is a methodology used to structure and analyze complex issues by leveraging expert knowledge. Because ISM derives insights from a small number of experts within a specific system, the expertise and credibility of panel members are critical.

In alignment with previous ISM studies in the health care domain [18-20] and consistent with the objective of systematizing practical knowledge in clinical settings, an expert panel of RDNs was convened at a medical institution in Japan, where the study was conducted. Participation was entirely voluntary, and eligible individuals were invited based on predefined eligibility criteria.

The eligibility criteria for the expert panel were as follows:

- At least 10 years of clinical experience as an RDN in medical institutions
- Ongoing involvement in routine nutritional counseling, with a workload of more than 10 counseling sessions per week
- Engagement in the training and education of dietetic students at accredited educational institutions

On the basis of these criteria, 3 RDNs with 19, 14, and 12 years of clinical experience participated in the study. The purpose and procedures of the study were explained in advance, and all participants voluntarily engaged in the structured consensus-building process as part of professional collaboration.

### Extraction of Elements and Procedure

A structured expert consensus method was adopted to extract elements relevant to the nutritional guidance techniques. The procedure comprised the following steps:

- Brainstorming—each participant independently listed the elements they considered influential on nutritional guidance.
- Sorting and categorization—the extracted elements were written on individual sticky notes and displayed on a large board. Similar items were grouped, and category names were assigned to each group. The relationships among the groups were then visually organized using connecting lines.
- Consensus formation—under the facilitation of a moderator, the participants discussed and confirmed the validity of the groupings and interrelationships. Each step was conducted independently to minimize bias, and the final decisions were made after a third-party review.

### Definition of the Elements

Each element was assigned a clear and concise definition based on the NCP to eliminate ambiguity and promote shared understanding among professionals.

### Statistical Analysis

The structural relationships among the elements were analyzed in accordance with the standardized ISM procedures as follows:

- Development of the adjacency matrix—all elements were arranged in a square matrix with element *i* on the vertical axis and element *j* on the horizontal axis. If element *i* influenced element *j*, then the matrix entry *a*_*ij*_ was set to 1; otherwise, it was set to 0. This resulted in the formation of the adjacency matrix A (Table S1 in Multimedia Appendix 1).
- Derivation of the reachability matrix—the identity matrix I was added to the adjacency matrix A to obtain matrix B. By exponentiating B, the reachability matrix T was calculated (Table S2 in Multimedia Appendix 1).
- Hierarchy level determination—for each element, a reachability set (R) and an antecedent set (Q) were determined. Elements satisfying the condition R ∩ Q = R were assigned to the highest hierarchical level. This process was repeated to classify all elements into hierarchical levels, which were then visualized in a structured diagram.

All analyses were conducted using College Analysis (version 8.5) [21].

### Independent Third-Party Review

To further enhance reproducibility and minimize bias, an independent third-party review was conducted for the ISM procedure and outputs. The reviewer was a researcher with more than 20 years of experience in the field of medical informatics who did not participate in the selection of candidates or the expert panel sessions. The review assessed (1) the comprehensiveness of the 14 elements and their operational definitions with reference to the NCP, (2) the audit trail from the original brainstorming records to the grouped categories and final elements, (3) the internal consistency of the pairwise comparisons and the resulting adjacency matrix, (4) the reproducibility of the reachability matrix and level assignments, and (5) the validity of the final hierarchical diagram. No major discrepancies requiring changes in element membership or hierarchy levels were identified, and only minor wording adjustments were recommended and implemented.

### Ethical Considerations

The Teine Keijinkai Hospital Ethics Committee of the authors’ affiliated institution determined that this study was not subject to ethical review under Japan’s Ethical Guidelines for Medical and Biological Research Involving Human Subjects, as it did not involve the collection of information on individual participants (ethical review status TKH-EC No. A1-3-025298).

## Results

This study identified 14 elements that influence nutritional guidance techniques and their definitions (Table 1).

**Table 1.** The 14 elements influencing nutritional guidance techniques and their definitions.

Element	Definition
Food, nutrition, and cooking knowledge	Correct recognition and understanding related to food, nutrition (including meals, snacks, enteral and parenteral nutrition, and supplements), and cooking
Clinical experience	Experience addressing nutritional problems in patients as a clinical dietitian
Learning opportunities	Time spent acquiring knowledge and skills outside of routine clinical practice
Multidisciplinary collaboration	Consulting, referring to, and coordinating with other health care professionals and institutions involved in nutritional management
Information gathering	Gathering clinical data and facts about patients whose nutritional status is presumed to be affected
Clinical knowledge	Correct recognition and understanding of clinical medicine and understanding of the patient’s condition
Communication skills	Ability to build good relationships with patients and various professionals
Physical assessment	Evaluating physical signs and symptoms in patients
Nutritional diagnosis	On the basis of nutritional assessment, comprehensively determining the nutritional status of the patient and identifying nutritional issues
Understanding of patient background	Understanding and respecting the patient’s social and mental conditions
Motivating	The psychological process that encourages a patient to start taking action toward a goal and reach that goal
Nutritional intervention plan	Intervention contents to improve and solve nutritional issues according to the individual needs of patients
Educational materials	Materials and media used to structure an educational curriculum aimed at facilitating mutual understanding between the dietitian and learners
Counseling techniques	Techniques used by clinical dietitians to assist patients using psychological methods from a professional standpoint

**Figure 1.**
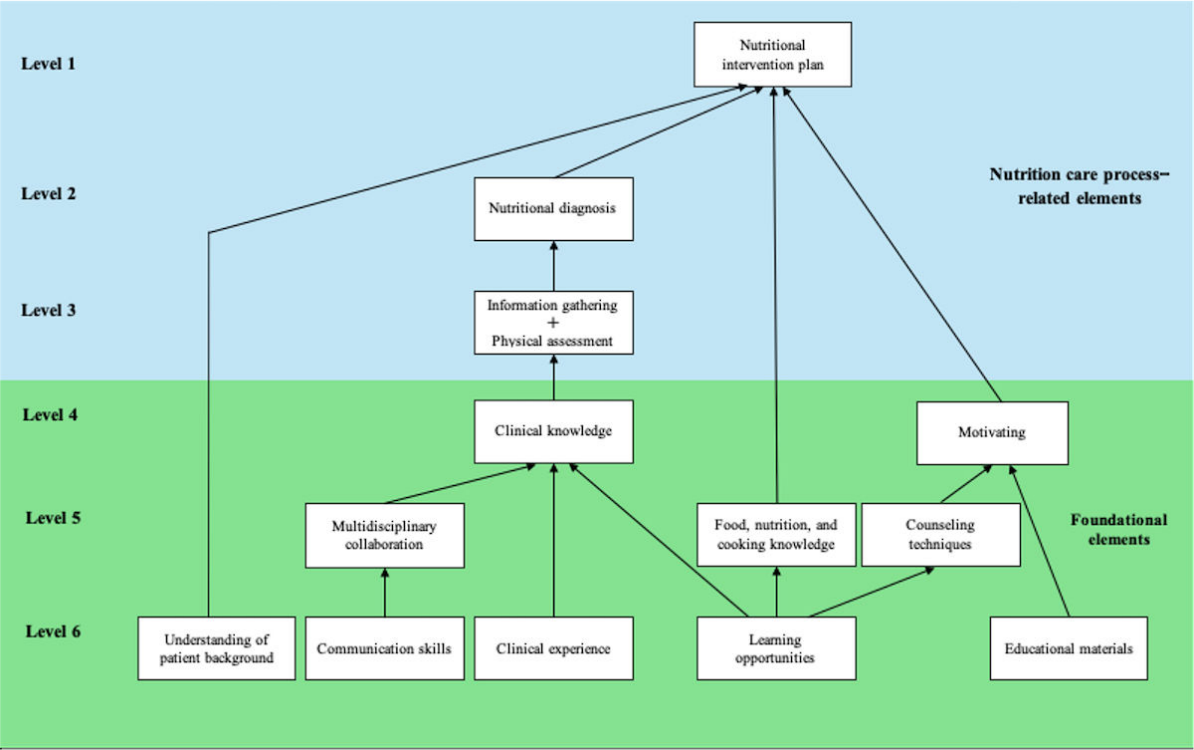
Hierarchical structure of 14 elements influencing nutritional guidance techniques identified using interpretive structural modeling.

Furthermore, a 6-level hierarchical structure based on these elements was obtained (Figure 1).

The 3 upper levels consisted of NCP-related elements. At the top level (level 1) was “nutritional intervention plan,” followed by “nutritional diagnosis” at level 2. Level 3 included “information gathering” and “physical assessment.” “Clinical knowledge” and “motivating” were placed at level 4. Level 5 comprised “food, nutrition, and cooking knowledge,” “counseling techniques,” and “multidisciplinary collaboration.” At the base level (level 6), “understanding of patient background,” “communication skills,” “clinical experience,” “learning opportunities,” and “educational materials” were located.

Figure 1 illustrates the 6-level hierarchical framework consisting of 14 elements that influence nutritional guidance techniques. Levels 1 to 3 represent higher-order elements related to the NCP, whereas levels 4 to 6 represent foundational elements that underpin these upper-level competencies.

## Discussion

### Principal Findings

This study is the first to systematically visualize both the components and the hierarchical structure of nutritional guidance techniques in clinical settings using ISM. A total of 14 elements that influence dietitians’ practical skills were extracted by 3 experienced registered dietitians, and the interrelationships of these elements were structured into 6 hierarchical levels. The findings reveal that nutritional guidance is not a linear sequence of procedures but a multilayered construct formed through the integration of knowledge, clinical experience, interpersonal skills, and contextual factors.

While the NCP model offers a procedural framework focused on standardizing nutrition care [6], this study offers a new perspective by capturing the interrelationships among the technical elements underpinning nutritional guidance techniques in clinical settings.

### Comparison With Previous Studies and Interpretation

All 14 elements identified in this study correspond closely to the essential competencies outlined in the most recent standards of practice and standards of professional performance (SOPP) by the Academy of Nutrition and Dietetics for RDNs [10-13], supporting the content validity of the results.

Specifically, “nutritional intervention plan,” “nutritional diagnosis,” “information gathering,” and “physical assessment” align directly with the core components of the NCP and are described as the foundational practical skills required for all dietitians. In addition, “clinical knowledge” and “clinical experience” are recognized as crucial for advanced practice, particularly for managing complex cases and making high-level judgments. “Counseling techniques,” including reflective listening and goal setting, are emphasized as behavior change strategies rooted in communication science.

The importance of “motivating” is underscored by its alignment with the principles of motivational interviewing, which aims to enhance patient engagement through individualized support. “Food, nutrition, and cooking knowledge” is fundamental for providing culturally appropriate and practical dietary advice. “Multidisciplinary collaboration” is considered a key driver of patient-centered care, requiring mutual understanding of roles across health care professionals.

“Understanding of patient background” and “communication skills” are regarded as indispensable competencies for providing support from a comprehensive perspective that encompasses cultural background, health literacy, and social factors. “Learning opportunities” are positioned in the SOPP as elements of self-development and professional growth that continuously enhance the expertise of registered dietitians, with expectations for engagement in professional certification and the training of future practitioners. Furthermore, “educational materials” are intended to facilitate patient understanding and promote behavior change. The role of RDNs is emphasized in selecting and using educational resources that are culturally and linguistically appropriate as well as visually accessible to support the delivery of patient-centered care.

The 14 elements extracted in this study corresponded closely to many of the competencies described in the standards of practice and SOPP; however, certain components, such as leadership, policy advocacy, and advanced research skills, were not included. While these skills are professionally important, they may be less directly observable in the context of daily clinical nutrition counseling. Ethical principles were also raised but were not retained as an independent element as they are regarded as a fundamental premise of professional practice for registered dietitians, as outlined in the code of ethics of the Japan Dietetic Association [22] and the 2018 Academy of Nutrition and Dietetics and Commission on Dietetic Registration Code of Ethics for the Nutrition and Dietetics Profession [23]. Therefore, the ISM model presented in this study does not aim to comprehensively reproduce all NCP-related competencies but, rather, to provide a practical framework focusing on the core techniques of clinical nutrition counseling.

### Structural Interpretation

The hierarchical positioning of “nutritional intervention plan,” “nutritional diagnosis,” “information gathering,” and “physical assessment” at the upper levels of the structure suggests that effective nutritional guidance is critically dependent on the proper implementation of the NCP. Among them, “nutritional intervention plan,” located at the topmost level, represents the culmination of the entire process and serves as the decisive element determining the success of dietary intervention. This is consistent with the theoretical foundation of the NCP [6], which emphasizes the structured and sequential application of care processes as essential to high-quality nutrition counseling.

In contrast, the lower-level elements—including “communication skills,” “clinical experience,” “learning opportunities,” “educational materials,” and “understanding of patient background”—represent the foundational competencies that support the execution of the upper-layer techniques. These elements emerged as enabling factors, suggesting that improvements in knowledge, skills, and environmental support can exert cascading effects on the quality of higher-order nutritional practices.

### Formation of Clinical Knowledge and the Role of Learning Opportunities

While prior studies have noted that nutritional guidance is influenced by the dietitian’s knowledge and experience [10], our analysis further clarifies that “clinical knowledge” is shaped not only by experience but also through “multidisciplinary collaboration” and “learning opportunities.” Engagement with other health care professionals provides exposure to interdisciplinary knowledge; enriches perspectives; and, ultimately, enhances clinical competence and service quality [24]. Brody et al [25] also found that advanced-level RDNs emphasize communication with patients, families, and the health care team as a core aspect of their work.

Furthermore, Dart et al [14] emphasized that interpersonal communication and lifelong learning are the central components of dietitian professionalism. In this study, “learning opportunities” was shown to influence “clinical knowledge,” “food, nutrition, and cooking knowledge,” and “counseling techniques,” reinforcing the notion that continuing education is essential for acquiring and maintaining advanced skills in nutrition counseling.

### Understanding Patient Background and Its Contribution to Person-Centered Care

Importantly, “understanding of patient background” emerged as a distinct and independent component of nutritional guidance, separate from “clinical knowledge.” Lövestam et al [26] pointed out that patients’ circumstances do not always fit neatly within the predefined structure of the NCP. Brody et al [27] similarly emphasized that effective practice by RDNs requires a deep understanding of patients’ complex backgrounds; delivery of person-centered care; and individualized, comprehensive support. Soguel et al [28] argued that integrating contextual information into care enhances quality and bridges the gap between knowledge and practice. In alignment with this, Holdoway et al [29] demonstrated that focusing on patients’ values, priorities, and needs significantly improves the outcomes of nutritional interventions. These findings reinforce the idea that understanding the social, cultural, and psychological context of patients is indispensable for delivering effective nutritional guidance.

In addition, the element “understanding of patient background” encompasses the recognition of social determinants of health. Yoshikawa et al [30] demonstrated that dietary-specific social support significantly influences eating behaviors. Accordingly, consideration of social determinants of health and related social support should be explicitly integrated when assessing patient background and planning nutritional interventions.

### Technical and Environmental Elements Supporting Professional Competence

The bottom level of the structure included both technical competencies to be acquired by dietitians and contextual elements that influence practice. Specifically, “understanding of patient background,” “communication skills,” and “clinical experience” are competencies to be developed, whereas “learning opportunities” and “educational materials” represent environmental enablers.

Postgraduate education in clinical settings is vital for maintaining and advancing professional competencies. Both Vogelzang and Roth-Yousey [31] and Palermo et al [32] stressed the need for continuous self-development, knowledge acquisition, and technical enhancement throughout the dietitian’s career. Dart et al [14] highlighted “lifelong learning” as a defining theme of professionalism in dietetics, emphasizing the importance of reflective practice—evaluating one’s own performance and taking action toward improvement.

Moreover, as Lövestam et al [33] noted, support from supervisors and colleagues is also a key factor influencing clinical practice. Organizational backing is crucial, particularly in busy clinical settings, where dietitians must secure time and resources for ongoing learning and skill updates. The combination of self-directed effort and structured institutional support is essential for sustained professional growth.

### Implications for Education and Practice

The hierarchical model developed in this study may serve as a practical framework for designing clinical education curricula and training programs for dietitians. For example, beginner-level training could prioritize foundational competencies at level 6, such as “communication skills” and “understanding of patient background.” Instruction could then gradually progress toward higher-level competencies such as “diagnosis” and “intervention planning.” This stepwise approach could enhance both comprehension and skill acquisition. Furthermore, this model may inform revisions of existing professional standards and assessment tools by offering a structured basis for evaluating clinical competence in nutrition guidance.

### Limitations and Future Directions

Several limitations must be acknowledged. First, the model was developed based on insights from a limited number of expert participants, which may affect its generalizability. However, the ISM approach emphasizes depth of expertise over sample size, and expert selection in this study adhered to methodological recommendations. Future research should further validate and refine the model through Delphi methods and multisite collaboration to ensure its robustness across diverse settings.

Second, although this study was conducted at a single health care institution, the use of internationally standardized NCP elements suggests that the findings may be applicable to other contexts. Furthermore, refinement and validation of the model may contribute to the improvement of dietitian education and clinical training programs. In particular, comparing novice and expert dietitians could help clarify developmental differences in competencies and guide the systematic design of competency-based curricula.

Finally, while ISM identifies the presence or absence of relationships among elements, it does not measure the strength of those relationships. Combining ISM with other analytical approaches may yield deeper insights into interdependencies among elements and support the development of more effective educational frameworks for dietitians.

### Conclusions

This study identified 14 elements influencing nutritional guidance techniques in clinical practice and systematically visualized their interrelationships as a 6-level hierarchical structure using ISM. The findings revealed that foundational competencies function as prerequisites for the development of higher-level NCP-based practice skills. The model developed in this study provides both theoretical and practical foundations for strengthening clinical education, establishing competency assessment frameworks, and standardizing nutrition counseling techniques, and it may serve as an initial framework contributing to the further advancement of nutrition guidance education.

## Supplementary material

10.2196/83185 Multimedia Appendix 1 The relational and reachability matrices derived from the interpretive structural modeling analysis of 14 elements influencing nutritional guidance techniques.

## Abbreviations

ISM: interpretive structural modeling
NCP: nutrition care process
RDN: registered dietitian nutritionist
SOPP: standards of professional performance
